# AMPK activation by ASP4132 inhibits non-small cell lung cancer cell growth

**DOI:** 10.1038/s41419-021-03655-2

**Published:** 2021-04-06

**Authors:** Ying-chen Xia, Jian-hua Zha, Yong-Hua Sang, Hui Yin, Guo-qiu Xu, Jie Zhen, Yan Zhang, Ben-tong Yu

**Affiliations:** 1 Department of Thoracic Surgery, The First Affiliated Hospital of Nanchang University, Nanchang, China; 2 Department of Thoracic Surgery, The Second affiliated Hospital of Soochow University, Suzhou, China; 3 Department of Thoracic Surgery, Qidong People’s Hospital, Qidong, China; 4 Department of Radiotherapy and Oncology, Affiliated Kunshan Hospital of Jiangsu University, Kunshan, China

**Keywords:** Targeted therapies, Non-small-cell lung cancer

## Abstract

Activation of adenosine monophosphate-activated protein kinase (AMPK) is able to produce significant anti-non-small cell lung cancer (NSCLC) cell activity. ASP4132 is an orally active and highly effective AMPK activator. The current study tested its activity against NSCLC cells. In primary NSCLC cells and established cell lines (A549 and NCI-H1944) ASP4132 potently inhibited cell growth, proliferation and cell cycle progression as well as cell migration and invasion. Robust apoptosis activation was detected in ASP4132-treated NSCLC cells. Furthermore, ASP4132 treatment in NSCLC cells induced programmed necrosis, causing mitochondrial p53-cyclophilin D (CyPD)-adenine nucleotide translocase 1 (ANT1) association, mitochondrial depolarization and medium lactate dehydrogenase release. In NSCLC cells ASP4132 activated AMPK signaling, induced AMPKα1-ACC phosphorylation and increased AMPK activity. Furthermore, AMPK downstream events, including mTORC1 inhibition, receptor tyrosine kinases (PDGFRα and EGFR) degradation, Akt inhibition and autophagy induction, were detected in ASP4132-treated NSCLC cells. Importantly, AMPK inactivation by AMPKα1 shRNA, knockout (using CRISPR/Cas9 strategy) or dominant negative mutation (T172A) almost reversed ASP4132-induced anti-NSCLC cell activity. Conversely, a constitutively active AMPKα1 (T172D) mimicked and abolished ASP4132-induced actions in NSCLC cells. In vivo, oral administration of a single dose of ASP4132 largely inhibited NSCLC xenograft growth in SCID mice. AMPK activation, mTORC1 inhibition and EGFR-PDGFRα degradation as well as Akt inhibition and autophagy induction were detected in ASP4132-treated NSCLC xenograft tumor tissues. Together, activation of AMPK by ASP4132 potently inhibits NSCLC cell growth in vitro and in vivo.

## Introduction

Lung cancer accounts for over 13% of all new cancer diagnoses^1,2^. In the United States alone, an estimated of 228,820 adults will be diagnosed with lung cancer each year^1,2^. Of which non-small cell lung cancer (NSCLC) constitutes close to 85% of all lung cancers^1,2^. NSCLC main subtypes include adenocarcinoma, squamous cell carcinoma, and large cell carcinoma^1,2^. Due to the lack of specific symptoms and ineffectiveness of current therapies, NSCLC prognosis is far from satisfactory. The five-year overall survival (OS) for stage I NSCLC is 47%, stage II is 30%, stage III is close to 10%, and stage IV is less than 1%^1,2^.

Adenosine monophosphate-activated protein kinase (AMPK) is a highly-conserved eukaryotic protein kinase, playing an essential role in cellular energy homeostasis^3–5^. With the cellular energy decreasing, activated AMPK is able to promote glucose and fatty acid uptake and oxidation^3–5^. AMPK is composed of α, β, and γ subunits^3–5^. AMPK can be activated with AMP binding or by phosphorylation at α subunit’s Thr-172 residue^6^. One of primary AMPK kinases is the tumor suppressor protein kinase LKB1^7^. Exonic and whole LKB1 gene deletions are presented in Peutz–Jeghers syndrome with a strong tendency of developing cancer^8,9^. In addition, somatic mutations and inactivation of LKB1 are associated with lung cancers^10^. Gill et al., observed that loss of heterozygosity or homozygous deletion of LKB1 gene was occurred in the majority of NSCLCs^10^. Interestingly, Hui et al., have shown that AMPKα1 expression levels are significantly higher in NSCLC tumor tissues^11^. These results implied that AMPK cascade could be an important therapeutic target of NSCLC.

Activation of AMPK could lead to metabolic tumor suppression due to energy metabolism regulation, metabolic checkpoint enforcement and growth inhibition^12^. AMPK activators are potential therapeutic candidates for cancer treatment^12–16^. Indeed, forced activation of AMPK signaling cascade, using pharmacological or genetic strategies, could exert potent anti-NSCLC cell activity. Chen et al., have shown that chrysin activated AMPK signaling cascade to cause growth inhibition and apoptosis in A549 NSCLC cells^17^. Conversely, AMPK inhibition or silencing largely attenuated chrysin-induced anti-A549 cell activity^17^. Wei et al. reported that cordycepin, by activating AMPK signaling, induced apoptosis in drug-resistance NSCLC cells^18^. Moreover, AMPK activation by metformin inhibited NSCLC cell growth and enhanced radiation sensitivity^19^. Furthermore, AMPK cascade activation by Circular RNA circHIPK3 induced autophagic death in NSCLC cells^20^.

Although activation of AMPK could exert profound anti-NSCLC cell activity^17–20^. The traditional AMPK activators, including AICAR and metformin, were often utilized at extreme high concentrations (at mM concentrations in vitro)^14,21,22^. Furthermore, these known AMPK activators could induce AMPK-independent toxicities^14,21,22^. Recent studies have developed ASP4132 as an orally active and potent AMPK activator with an EC50 of 18 nM^23,24^. This novel AMPK activator has displayed potent and selective cell growth inhibitory activity against breast cancer cell lines in vitro^24^. Furthermore, oral administration of ASP4132 has shown potent in vivo anti-tumor efficacy in a MDA-MB-453 xenograft mouse model^24^. Metabolic stability and in vivo animal pharmacokinetics (PK) profiles of ASP4132 are favorable^24^. Its aqueous solubility at gastrointestinal pH is also acceptable^23,24^. We therefore hypothesized that activation of AMPK by ASP4132 could possibly exert potent anti-NSCLC cell activity.

## Materials and methods

### Chemicals and reagents

ASP4132 was purchased from MCE China (Beijing, China). ASP4132 powder was first dissolved in DMSO to 100 mM stock solution. For in vitro studies, ASP4132 stock solution was further dissolved in PBS before adding to cell culture medium. For in vivo studies, ASP4132 stock solution was dissolved in 40% PEG400-5% Tween-80 saline solution and applied by oral gavage. Antibodies of p-AMPKα1 (Thr172, #2531), AMPKα1 (#2532), acetyl-CoA Carboxylase (ACC, #3662), p-ACC (Ser79, #3661), p-Akt (Thr308, #5110), Akt1/2 (#2967), GAPDH (glyceraldehyde-3-phosphate dehydrogenase, #5174), p70 S6 Kinase (S6K1 #9202), p-S6K1 (Thr389, #9205), p-S6 Ribosomal Protein (Ser235/236, #2211), EGFR (#2232), PDGFRα (#4547), cleaved caspase antibody sampler Kit (#9929), Erk1/2 (#4695), and β-Tubulin (#2146) were purchased from Cell Signaling Technologies (Beverly, MA). The autophagy antibody kit was purchased from Cell Signaling Technologies (#4445) as well. The anti-adenine nucleotide translocase 1 (ANT1) antibody (ab102032) was purchased from Abcam China (Shanghai, China). Antibodies for cyclophilin-D (CyPD, sc-137136) and p53 (sc-126) were provided by Santa Cruz Biotech (Santa Cruz, CA). All reagents for cell culture, including fetal bovine serum (FBS) and antibiotics, were provided by Hyclone (Logan, UT). From Sigma-Aldrich Chemicals Co. (St. Louis, Mo) puromycin, polybrene, z-DEVD-fmk, z-VAD-fmk and all other chemicals were obtained. Shanghai Genechem Co. (Shanghai, China) synthesized and verified all primers, sequences, constructs and virus, unless otherwise mentioned. JC-1, EdU, DAPI, TUNEL and CellROX fluorescence dyes, Annexin V, propidium iodide (PI) and PCR reagents were purchased from Thermo-Fisher Invitrogen (Shanghai, China).

### Cell culture

NSCLC cell lines, NCI-H1944 and A549, were purchased from Shanghai Institute of Biochemistry and Cell Biology (Shanghai, China). Cells were cultured in DMEM together with 10% FBS. Primary human NSCLC cells, derived from three written-informed consent NSCLC patients (pNSCLC-1/-2/-3), as well as BEAS-2B lung epithelial cells and primary lung epithelial cells, were provided by Dr. Jiang^25,26^, and cells were cultured as described previously^25,26^. No patients were treated with any additional therapies before surgery. The protocols of using human cells were approved by the Ethics Committee of Nanchang University (NCUBR099), in according to Declaration of Helsinki. All cells utilized in this study were subjected to mycoplasma and microbial contamination examination every two months. Authentication by STR profiling, population doubling time, and cell morphology were routinely checked to verify their genotypes.

### Cell viability

NSCLC cells or lung epithelial cells were seeded into 96-well plates (at 3 × 10^5^ cells/mL). Following the applied treatment CCK-8 was added to each well. CCK-8 absorbance (optical density, OD) was detected by a plate reader at the test wavelength of 550 nm.

### “Transwell” assays

Following the applied treatment, NSCLC cells were trypsinized, re-suspended into serum-free medium and added on the upper surfaces of Transwell chambers (BD Biosciences, Shanghai, China). The lower chambers were filled with complete medium with 10% FBS. After 24 h of migration, the migrated cells in the lower chambers were fixed, stained and counted. Invasion assays were conducted using the same protocol except the “Transwell” inserts were pre-coated with Matrigel (Sigma).

### EdU staining

NSCLC cells were initially seeded into 12-well plates at 3 × 10^4^ cells per well and were allowed to adhere overnight and subjected to treatments accordingly. Afterwards, cells were washed, fixed and stained with EdU for 2 h. Cell nuclei were co-stained with DAPI. For each condition at least 1200 nuclei from five random microcopy views (1 × 100) were counted to calculate EdU-positive nuclei ratio (EdU/DAPI × 100%).

### Clonogenic assays

NSCLC cells were initially seeded into six-well plates (10,000 cells per well) and maintained at the ASP4132-containing complete medium. After ten days colonies were fixed, stained and counted.

### Cell cycle studies

NSCLC cells were seeded at 0.4 × 10^6^ cells per well and subjected to applied treatment. Afterwards, cells were washed in PBS, trypsinized, centrifuged and resuspended in 1 mL of 95% ethanol and stored at −20 °C for 24 h. Cells were then centrifuged and resuspended in 1 mL of propidium iodide (PI) staining solution, and eventually were tested under a FACS-calibur flow cytometry (Beckman-Coulter, Shanghai, China). Data analyses were performed via an Expo32 ADC v1.1c software.

### Annexin-V assay

NSCLC cells were seeded at 1 × 10^6^ cells per mL and subjected to applied treatment. Cells were then washed, resuspended and fixed. Afterwards, cells were incubated with PI (10 μg/mL) and Annexin V (10 μg/mL), and were examined by the FACS-calibur flow cytometry (Beckman-Coulter). Annexin V-positive cells were gated as apoptotic cells and its ratio was recorded.

### Apoptotic nuclei assay

NSCLC cells were seeded onto a glass cover-slip and subjected to applied treatment. Cells were then rinsed in PBS and co-stained with Hoechst-33342 and TUNEL dyes. Apoptotic nuclei presented with condensed DNA and fragmented nuclei staining (certain apoptotic nuclei were positive with TUNEL staining). For each condition at least 1200 nuclei from five random microcopy views (1 × 100) were counted to calculate apoptotic nuclei ratio.

### Western blotting

Cells and tissues were incubated with the lysis buffer (as described^18^). From each treatment protein lysates (40 μg per lane) were resolved by 10–12% SDS-PAGE gels and transferred to PVDF membranes (Millipore, Shanghai, China). Membranes were incubated sequentially in PBST containing 10% non-fat milk, followed by incubation with primary antibody (overnight at 4 °C) and secondary antibody (2 h at room temperature). An ECL Western Blotting Substrate Kit (Abcam, Shanghai, China) was utilized to visualize the targeted protein band. Image J software from NIH website was utilized for data quantification.

### Quantitative real time-PCR (qPCR) assay

Total cellular RNA was extracted via TRIzol reagents (Thermo-Fisher Invitrogen) and was reverse-transcripted. The ABI Prism 7900 Fast Real-Time PCR system was utilized for qPCR assays. The product melting temperature was calculated. Quantification of targeted mRNAs was through the 2^−∆∆*C*t^ method, using *GAPDH* as an internal control. The mRNA primers for *EGFR*, *PDGFRα* and GAPDH were from Dr. Chen at Jiangsu University^27^.

### Caspase-3 activity

Following treatment the hypotonic cell lysis buffer^18^ was utilized to extract cytosolic proteins. The protein lysates (20 μg per treatment) were added to the caspase assay buffer^18^ containing the caspase-3 substrate (Calbiochem, Darmstadt, Germany). After 2 h incubation, released 7-amido-4-(trifluoromethyl)coumarin (AFC) was quantified through a Fluoroskan system (Thermo-Labsystems, Helsinki, Finland)^18^.

### Mitochondrial immunoprecipitation (Mito-IP)

The detailed procedures of Mito-IP were reported early^28,29^. In brief, mitochondrial lysates were obtained from NSCLC cells with applied treatment^30,31^. Lysates (500 μg per condition) were pre-cleared and incubated with anti-cyclophilin-D (CypD) antibody (Santa Cruz Biotech). CypD-immunoprecipitated proteins, adenine nucleotide translocator-1 (ANT1) and p53, were captured and tested by Western blotting.

### Mitochondrial depolarization

JC-1 is able to accumulate in mitochondria as monomers in cells undergoing mitochondrial membrane potential (MMP) reduction. With mitochondria depolarization JC-1 will then emit green fluorescence (490 nm)^32^. The detailed protocols of JC-1 assaying of mitochondrial depolarization were described early^33^. JC-1 fluorescence images, merging both the green fluorescence channel and the red fluorescencence channel, were presented.

### AMPKα1 shRNA

NSCLC cells were seeded into six-well plates at 50% confluence and treated with AMPKα1 shRNA lentiviral particles (Sigma-Aldrich, 20 μL per well). NSCLC cells were further cultured for 48 h. Afterwards, cells were cultured in complete medium containing puromycin (2.5 μg/mL). After six passages AMPKα1 expression in the stable cells was examined by Western blotting assays.

### AMPKα1 KO

The CRISPR/Cas9 AMPKα1-KO construct was provided by Dr. Pan at Shanghai Jiao Tong University School of Medicine^34^ and was transduced to pNSCLC-1 cells by Lipofectamine 3000. Transfected cells were distributed to 192-well plates and subjected to AMPKα1-KO screen. The achieved single cells were further selected by puromycin to achieve stable AMPKα1-KO cells.

### AMPK mutation

The constitutively active AMPKα1 (T172D, caAMPKα1) adenoviral vector and the dominant negative AMPKα1 (T172A, dnAMPKα1) adenoviral vector, both containing GFP and puromycin selection gene, were provided by Dr. Wang at Soochow University^35^. NSCLC cells were seeded into six-well plates at 50% confluence and transfected with caAMPKα1 vector or dnAMPKα1 vector using Lipofectamine 3000 (Thermo-Fisher Invitrogen). Transfected cells with GFP were sorted by FACS and distributed into 192-well plates. Single stable cells were further selected by puromycin-containing medium. The empty vector was transfected by Lipofectamine 3000 (Invitrogen, Carlsbad, CA) to control cells.

### AMPK activity assay

Total cellular lysates were initially incubated with an anti-AMPKα1 antibody (Santa Cruz Biotech). AMPK activity was measured by using AMP-[γ-^32^P] ATP mixture and AMPK substrate SAMS (HMRSAMSGLHLVKRR) peptide. To stop the reaction the phosphocellulose paper was included in the kinase assay buffer. The AMPK radioactivity was examined by a scintillation counter.

### Light chain 3B (LC3B) staining

As reported^36,37^, NSCLC cells with applied treatment were fixed and incubated with anti-LC3B antibody (red fluorescence protein/RFP-conjugated, Genechem, Shanghai, China). LC3B RFP fluorescence puncta in the autophagic cells was visualized under a Leica microscope.

### NSCLC mouse xenograft assay

Five-week-old severe combined immunodeficient (SCID) mice (half male half female, 18.5–19.5 g in weights) were maintained under Animal Facility of Soochow University Medical School (Suzhou, China). Each mice was grafted with 3 × 10^6^ pNSCLC1 cells through *s.c*. injection to the right flank. Within three weeks when tumor volume reached 100 mm^3^ (labeled as “Day-0”) animals were randomly assigned into two groups, receiving either vehicle control or the applied ASP4132 administration (ten mice per group, *n* = 10). Tumor dimensions were measured by calliper every six days and volume was estimated as per: *V* = length × width × height × 0.5236. At experimental Day-7 and Day-14, one tumor of each group was extracted via surgery. Tumors were cut into small pieces and lysed in tissue lysis buffer (Biyuntian, Wuxi, China). Protein lysates were tested by Western blotting. All animal experiments were approved by Animal Ethics Board of Nanchang University (ID: NCUBR015).

### Statistical analysis

All in vitro experiments in this study were repeated five times with similar results obtained. Data were presented as mean ± standard deviation (SD, *n* = 5). Error bars stand for five replicated wells/dishes for each in vitro experiment. One-way ANOVA (plus Student-Newman-Keuls post hoc test) was carried out to determine statistical significance between multiple groups (SPSS23.0, SPSS Co. Chicago, CA). A two-tailed Student’s *t*-test (Excel 2007) was utilized when comparing difference between two specific groups. Values of *P* < 0.05 were considered as statistically different.

## Results

### ASP4132 treatment exerts potent anti-NSCLC cell activity

**Fig. 1:**
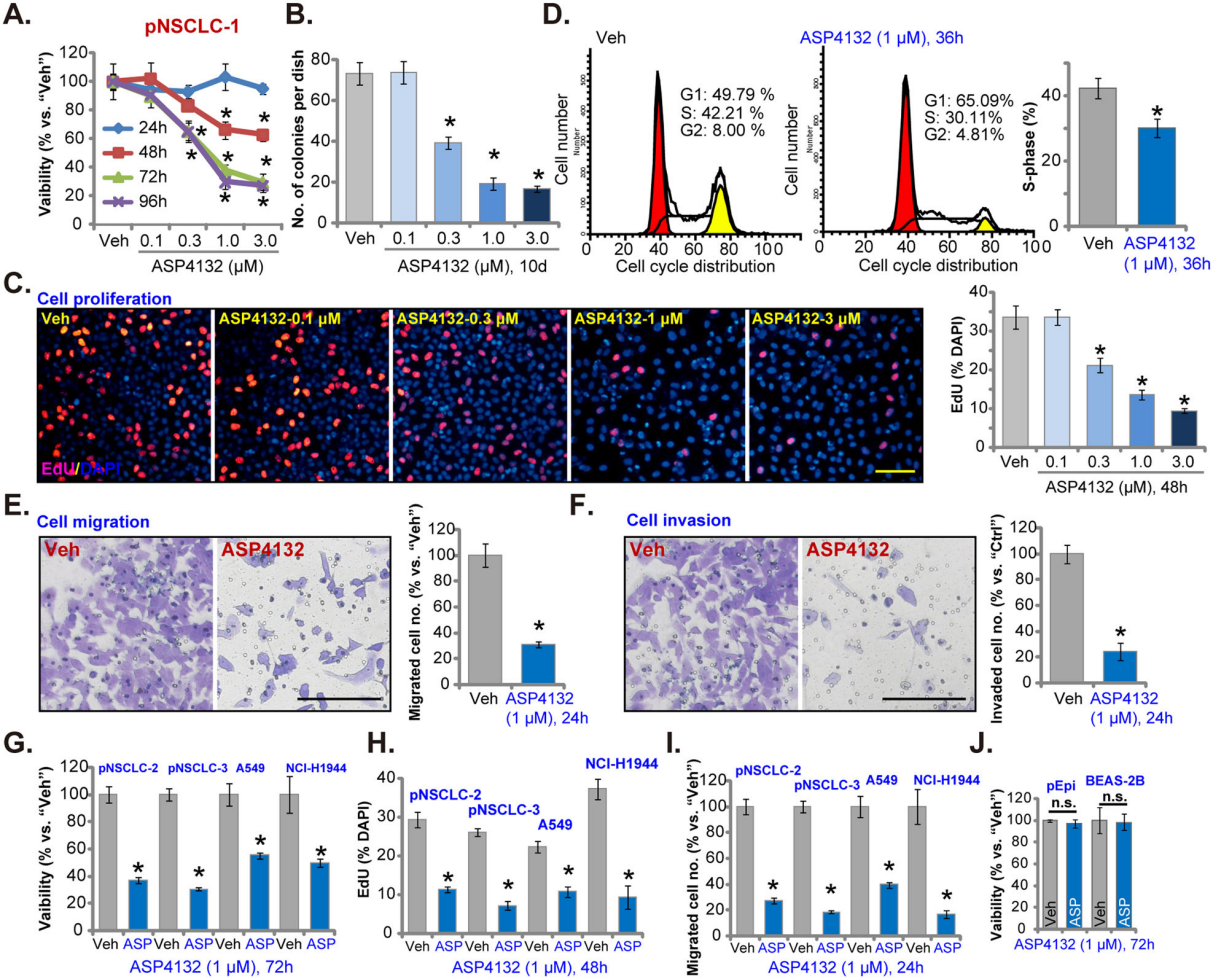
ASP4132 treatment exerts potent anti-NSCLC cell activity. Primary NSCLC cells that were derived from different patients (pNSCLC-1/-2/-3), the immortalized NSCLC cell lines (A549 and NCI-H1944), as well as primary (“pEpi”) and established (BEAS-2B) lung epithelial cells, were treated with ASP4132 at applied concentrations, cells were further cultured for applied time periods, cell viability (CCK-8 assay, **A**, **G**, **J**), colony formation (**B**), proliferation (EdU incorporation assay, **C**, **H**), cell cycle progression (**D**), as well as cell migration (**E**, **I**) and invasion (**F**) were tested by assays mentioned in the text, with results quantified. For all EdU assays in this study, five random views containing at least 1, 200 cells of each condition were included to calculate EdU ratio (% vs. DAPI). For all “Transwell” and “Matrigel Transwell” assays in this study, five random views of each condition were included to calculate the average number of migrated/invaded cells. “Veh” stands for the vehicle control (Saline). Data were presented as mean ± standard deviation (SD, *n* = 5). *p < 0.05 vs. “Veh” cells. “n.s.” stands for no statistical difference (**J**). The experiments were repeated five times with similar results detected. Scale bar = 100 μm (**C**, **E**, **F**).

The primary human NSCLC cells, pNSCLC-1 (as reported early^25,26^), were cultured in FBS (10%)-containing complete medium and treated with gradually-increased concentrations of ASP4132 (0.1–3.0 μM). CCK-8 assay was performed to test cell viability. Results in Fig. 1A showed that ASP4132 decreased pNSCLC-1 cell viability in a concentration-dependent manner. ASP4132, at 0.3–3.0 μM, was significant in inhibiting cell viability (Fig. 1A), but was ineffective at 0.1 μM (Fig. 1A). The AMPK activator also showed a time-dependent response in inhibiting pNSCLC-1 cell viability, as it required at least 48 h to exert a significant effect (Fig. 1A). In Fig. 1B the colony formation assay results displayed that ASP4132 (0.3–3.0 μM) significantly decreased the number of viable pNSCLC-1 cell colonies, further supporting its anti-survival activity. Testing cell proliferation, using EdU incorporation assay, demonstrated that ASP4132 dose-dependently decreased the ratio of EdU-positive nuclei in pNSCLC-1 cells (Fig. 1C). ASP4132 at 0.1 μM again failed to significantly inhibit colony formation (Fig. 1B) and EdU incorporation (Fig. 1C). Since 1 μM of ASP4132 potently inhibited cell viability (Fig. 1A), colony formation (Fig. 1B) and EdU incorporation (Fig. 1C), this concentration was selected for the following studies.

The PI-FACS assay was carried out to examine cell cycle progression. In ASP4132-treated pNSCLC-1 cells, G1-phase cells were increased, while S-phase cells were reduced (Fig. 1D). These results indicated that the AMPK activator disrupted cell cycle progression and induced G1-S arrest in pNSCLC-1 cells (Fig. 1D). “Transwell” assays were utilized to test cell migration. Results showed that ASP4132 (1 μM) treatment significantly decreased the number of migrated pNSCLC-1 cells (Fig. 1E). In addition, pNSCLC-1 cell invasion, tested by “Matrigel Transwell” assays, was inhibited by ASP4132 treatment as well (Fig. 1F). Notably, for the “Transwell” assays pNSCLC-1 cells were treated with ASP4132 for only 24 h with no significant viability reduction detected (Fig. 1A).

Next the potential effect of ASP4132 was tested in other NSCLC cells. The primary NSCLC cells that were derived from two other patients, pNSCLC-2 and pNSCLC-3, as well as immortalized NSCLC cell lines (A549 and NCI-H1944), were cultured. With ASP4132 (1 μM) treatment, CCK-8 viability was significantly decreased in the primary and established NSCLC cells (Fig. 1G). Furthermore, in the NSCLC cells the AMPK activator inhibited cell proliferation and migration, which were tested by EdU incorporation (results quantified in Fig. 1H) and “Transwell” (results quantified in Fig. 1I) assays, respectively. In primary lung epithelial cells (“pEpi”) and established BEAS-2B epithelial cells^38^, ASP4132 (1 μM, 72 h) treatment failed to significantly inhibit cell viability (Fig. 1J), indicating a cancer cell specific effect by the AMPK activator. Together, in NSCLC cells ASP4132 potently inhibited cell viability, proliferation and cell cycle progression as well as cell migration and invasion.

### ASP4132 induces apoptosis activation in NSCLC cells

**Fig. 2:**
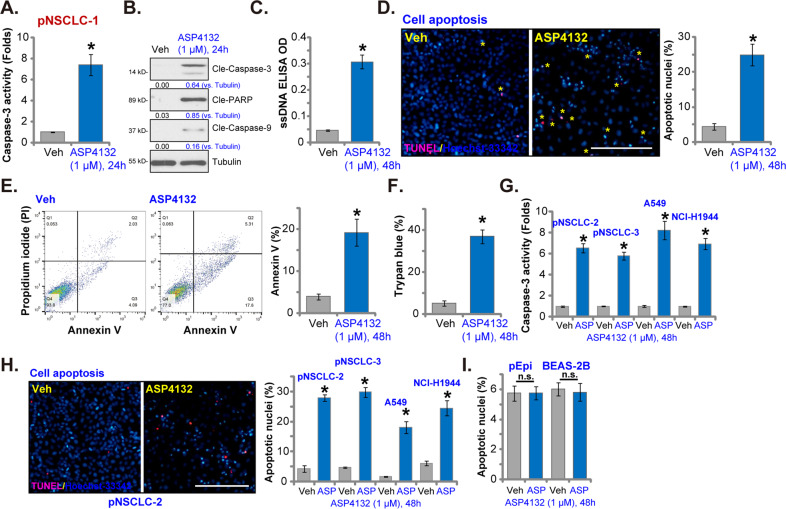
ASP4132 induces apoptosis activation in NSCLC cells. Primary NSCLC cells that were derived from different patients (pNSCLC-1/-2/-3), the immortalized NSCLC cell lines (A549 and NCI-H1944), as well as primary (“pEpi”) and established (BEAS-2B) lung epithelial cells, were treated with ASP4132 (1 μM) or the vehicle control; Cells were further cultured for applied time periods, caspase activation (**A**, **B**, **G**), single strand DNA (ssDNA) contents (ELISA assays, **C**) and cell apoptosis (**D**, **E**, **H**, **I**) were tested by the assays mentioned in the text, with cell death examined by Trypan blue staining assays (**F**). For all apoptotic nuclei assays in this study, five random views with total 1, 200 cells of each condition were included to calculate the average apoptotic nuclei ratio (% vs. total nuclei). Expression of listed proteins was quantified and normalized to the loading control (**B**). “Veh” stands for the vehicle control (Saline). Data were presented as mean ± standard deviation (SD, *n* = 5). *p < 0.05 vs. “Veh” cells. “n.s.” stands for no statistical difference (**I**). The experiments were repeated five times with similar results detected. Scale bar = 100 μm (**D**, **H**).

Forced activation of AMPK, through genetic or pharmacological strategies, was able to provoke cancer cell apoptosis^17,39,40^. In Fig. 1 we showed that ASP4132 induced proliferation inhibition and cell cycle arrest, we next tested its effect on cell apoptosis. As shown ASP4132 (1 μM) treatment significantly increased caspase-3 activity in pNSCLC-1 cells (Fig. 2A). The AMPK activator induced robust cleavages of caspase-3, caspase-9 and poly (ADP-ribose) polymerase (PARP) in pNSCLC-1 cells (Fig. 2B). Single strand DNA (ssDNA) accumulation, indicating DNA breaks, was detected in ASP4132-treated cells (Fig. 2C). Importantly, the ratio of apoptotic nuclei was dramatically increased following ASP4132 treatment in pNSCLC-1 cells (Fig. 2D). The apoptotic nuclei showed condensed or fragmented nuclear (Hoechst-33342) staining (characteristic nuclei were labeled with yellow stars, Fig. 2D), of which some were also TUNEL-positive (Fig. 2D). To further confirm apoptosis activation, FACS assay results showed that ASP4132 significantly increased the number of Annexin V-positive cells (Fig. 2E). Increased trypan blue staining further confirmed the cytotoxic effect of the AMPK activator in pNSCLC-1 cells (Fig. 2F).

In other primary (pNSCLC-2 and pNSCLC-3) and established (A549 and NCI-H1944 lines) NSCLC cells, ASP4132 treatment (1 μM, 48 h) significantly increased caspase-3 activity (Fig. 2G) and the apoptotic nuclei ratio (Fig. 2H). In contrast, no significant apoptosis activation was detected in primary (“pEpi”) and established (BEAS-2B) lung epithelial cells with the same ASP4132 treatment (Fig. 2I), further supporting cancer cell specific response by the AMPK activator. These results clearly showed that ASP4132 activated apoptosis in NSCLC cells.

### ASP4132 activates programmed necrosis cascade in NSCLC cells

**Fig. 3:**
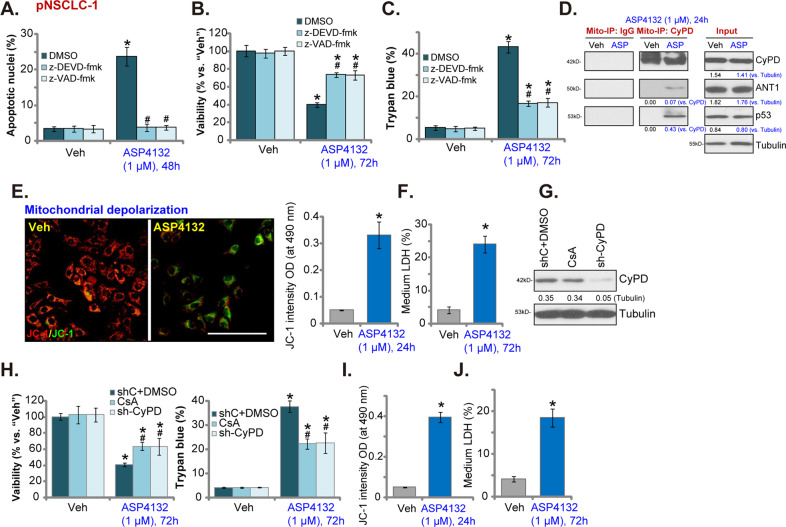
ASP4132 activates programmed necrosis cascade in NSCLC cells. Primary pNSCLC-1 cells were pretreated with the caspase-3 inhibitor z-DEVD-fmk (50 μM, 1 h pretreatment) or the pan caspase inhibitor z-VAD-fmk (50 μM, 1 h pretreatment), followed by ASP4132 (1 μM) treatment and cultured for applied time periods, cell apoptosis (apoptotic nuclei ratio, **A**), viability (CCK-8 OD, **B**) and cell death (Trypan blue positive cell ratio, **C**) were tested. Primary NSCLC cells, pNSCLC-1/-2, were treated with ASP4132 (1 μM) or the vehicle control, cells were further cultured for applied time periods, mitochondrial CyPD-p53-ANT1 association (Mito-IP, **D**), mitochondrial depolarization (by measuring JC-1 green monomers intensity, **E**, **I**) and cell necrosis (by measuring medium LDH contents, **F**, **J**) were tested. Expression of CyPD and Tubulin in stable pNSCLC-1 cells with CyPD shRNA lentiviral particles (sh-CyPD) or cyclosporin A (CsA, 5 μM, 24 h) treatment was shown (**F**). Control cells were treated with vehicle control plus scramble control shRNA lentiviral particles (“shC+DMSO”) (**G**); Cells were further treated with ASP4132 (1 μM) and cultured for 72 h, cell viability (CCK-8 OD) and cell death (Trypan blue positive cell ratio) were tested (**H**). Expression of listed proteins was quantified and normalized to the loading control (**D**, **G**). “Veh” stands for the vehicle control (Saline). Data were presented as mean ± standard deviation (SD, *n* = 5). *p < 0.05 vs. “Veh” cells. ^**#**^*p* < 0.05 vs^.^ “DMSO (0.2%)” pretreatment (**A**–**C**). ^**#**^*p* < 0.05 vs. “sh**C** + DMSO^**”**^ cells (**H**). The experiments were repeated five times with similar results detected. Scale bar = 100 μm (**E**).

To block apoptosis activation two caspase inhibitors, including the caspase-3 inhibitor z-DEVD-fmk and the pan caspase inhibitor z-VAD-fmk, were utilized. By recording apoptotic nuclei ratio, Fig. 3A, we showed that z-DEVD-fmk and z-VAD-fmk blocked ASP4132-induced apoptosis activation in pNSCLC-1 cells (Fig. 3A). However, the two only inhibited, but did not reverse, ASP4132-induced viability (CCK-8 OD) reduction (Fig. 3B) and cell death (Trypan blue ratio increase, Fig. 3C). These results suggested that ASP4132-induced cytotoxicity in NSCLC cells was not solely due to apoptosis activation.

Besides apoptosis, cancer cells can undergo programmed necrosis when facing various stimuli and anti-cancer agents^41,42^. Programmed necrosis is a mitochondria-dependent active cell death process^41,42^. Using the mitochondrial immunoprecipitation (“Mito-IP”) assay, we found that ASP4132 induced cyclophilin-D (CypD) association with p53 and adenine nucleotide translocator-1 (ANT1) in the mitochondria of pNSCLC-1 cells (Fig. 3D, “Mito-IP: CyPD”). It is a key initial step of programmed necrosis cascade activation^41,43,44^. Mitochondrial expression of CyPD, p53 and ANT1 was unchanged with ASP4132 treatment (Fig. 3D, “Input”).

Furthermore in ASP4132-treated pNSCLC-1 cells, profound mitochondrial depolarization was detected (Fig. 3E), evidenced by JC-1 green monomers accumulation in mitochondria^45^. These events were followed by LDH release to the medium (Fig. 3F), a characteristic marker of cell membrane rupture and cell necrosis. Therefore, ASP4132 induced mitochondrial CyPD-p53-ANT1 association (Fig. 3D), mitochondrial depolarization (Fig. 3E) and medium LDH release (Fig. 3F), suggesting activation of the programmed necrosis cascade (see studies testing the same cascade^43,46–49^).

To block programmed necrosis, we utilized a CyPD inhibitor cyclosporin A (CsA)^41,44^. Alternatively, CyPD shRNA lentiviral particles were added to pNSCLC-1 cells, and via puromycin selection stable cells were established (sh-CyPD cells). CyPD protein expression was silenced in sh-CyPD pNSCLC-1 cells (Fig. 3G). As shown CsA or CyPD shRNA inhibited ASP4132-induced viability (CCK-8 OD) reduction and cell death (Fig. 3H), suggesting that the programmed necrosis should also participate in ASP4132-induced anti-NSCLC cell activity. However, ASP4132-induced apoptosis activation in pNSCLC-1 cells was not affected by CsA or CyPD shRNA (Supplementary Fig. S1A). The two caspase inhibitors, z-DEVD-fmk and z-VAD-fmk, failed to attenuate ASP4132-induced medium LDH release (the necrosis indicator) in pNSCLC-1 cells (Supplementary Fig. S1B). In pNSCLC-2 cells ASP4132 treatment similarly induced mitochondrial depolarization (JC-1 green monomers intensity, Fig. 3I) and cell necrosis (medium LDH release, Fig. 3J). Collectively, these results confirmed that ASP4132 activated programmed necrosis cascade in NSCLC cells.

### ASP4132 activates AMPK signaling in NSCLC cells

**Fig. 4:**
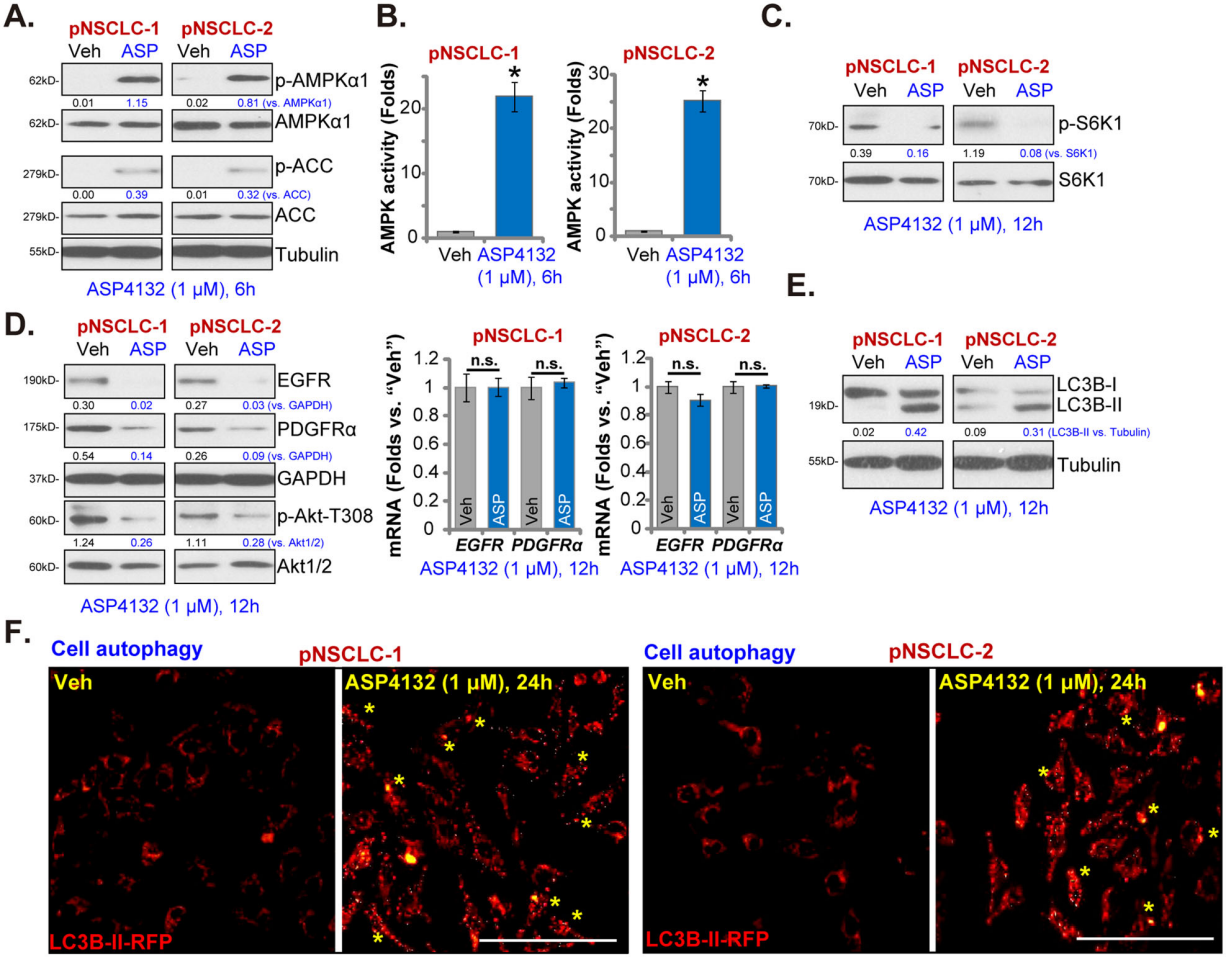
ASP4132 activates AMPK signaling in NSCLC cells. Primary NSCLC cells, pNSCLC-1/-2, were treated with ASP4132 (1 μM) or the vehicle control, cells were further cultured for applied time periods, expression of listed proteins in total cell lysates was tested by Western blotting assays (**A**, **C**, **D**, **E**); The relative AMPK activity was tested as well (**B**). List mRNAs were tested by qPCR assays (**D**). LC3B-II RFP (red fluorescence protein) puncta was examined (**F**). Expression of listed proteins was quantified and normalized to the loading control (**A**, **C**–**E**). “Veh” stands for the vehicle control (Saline). Data were presented as mean ± standard deviation (SD, *n* = 5). *p < 0.05 vs. “Veh” cells. “n.s.” stands for no statistical difference (**D**). The experiments were repeated five times with similar results detected. Scale bar = 100 μm (**F**).

ASP4132 is a novel AMPK activator^23,24^, we therefore tested its effect on AMPK signaling in NSCLC cells. Western blotting assay results, Fig. 4A, demonstrated that ASP4132 (1 μM) induced robust phosphorylation of AMPKα1 (Thr-172) and ACC (Ser79) in pNSCLC-1 and pNSCLC-2 cells. AMPKα1 and ACC protein expression was unchanged (Fig. 4A). In addition, the AMPK activity increased over twenty folds in ASP4132-treated NSCLC cells (Fig. 4B). These results indicated that ASP4132 activated AMPK signaling in NSCLC cells.

Activated AMPK is able to exert anti-cancer cell activity by regulating its targets, including mammalian target of rapamycin (mTOR) complex 1 (mTORC1) inhibition^50–52^, autophagy induction^52–54^ and degradation of various oncogenes^27,55^. We showed that phosphorylation of S6K1, the indicators of mTORC1 activation, was almost completely blocked by ASP4132 in pNSCLC-1 and pNSCLC-2 cells (Fig. 4C). A previous study by Chen et al., has demonstrated that AMPK activation is able to induce lysosomal translocation and degradation of PDGFR and EGFR^27^. We here found that protein levels of PDGFRα and EGFR were significantly decreased in ASP4132-treated NSCLC cells as well (Fig. 4D). *PDGFRα* and *EGFR* mRNA levels were however unchanged (Fig. 4D). Akt phosphorylation at Thr-308, the key downstream signal molecular of PDGFRα and EGFR, was also inhibited (Fig. 4D).

In addition, in pNSCLC-1 and pNSCLC-2 cells ASP4132 treatment induced LC3B-I conversion to LC3B-II (Fig. 4E), the characteristic marker of autophagy induction^56^. Furthermore, intense LC3B-II RFP (red fluorescence protein) puncta were accumulated in the cytosol of ASP4132-treated pNSCLC-1 and pNSCLC-2 cells (Fig. 4F), confirming autophagy induction. Collectively, these results showed that ASP4132 activated AMPK signaling, causing mTORC1 inhibition, PDGFRα-EGFR protein degradation, Akt inactivation, and autophagy induction in NSCLC cells.

### AMPK activation mediates ASP4132-induced anti-NSCLC cell activity

**Fig. 5:**
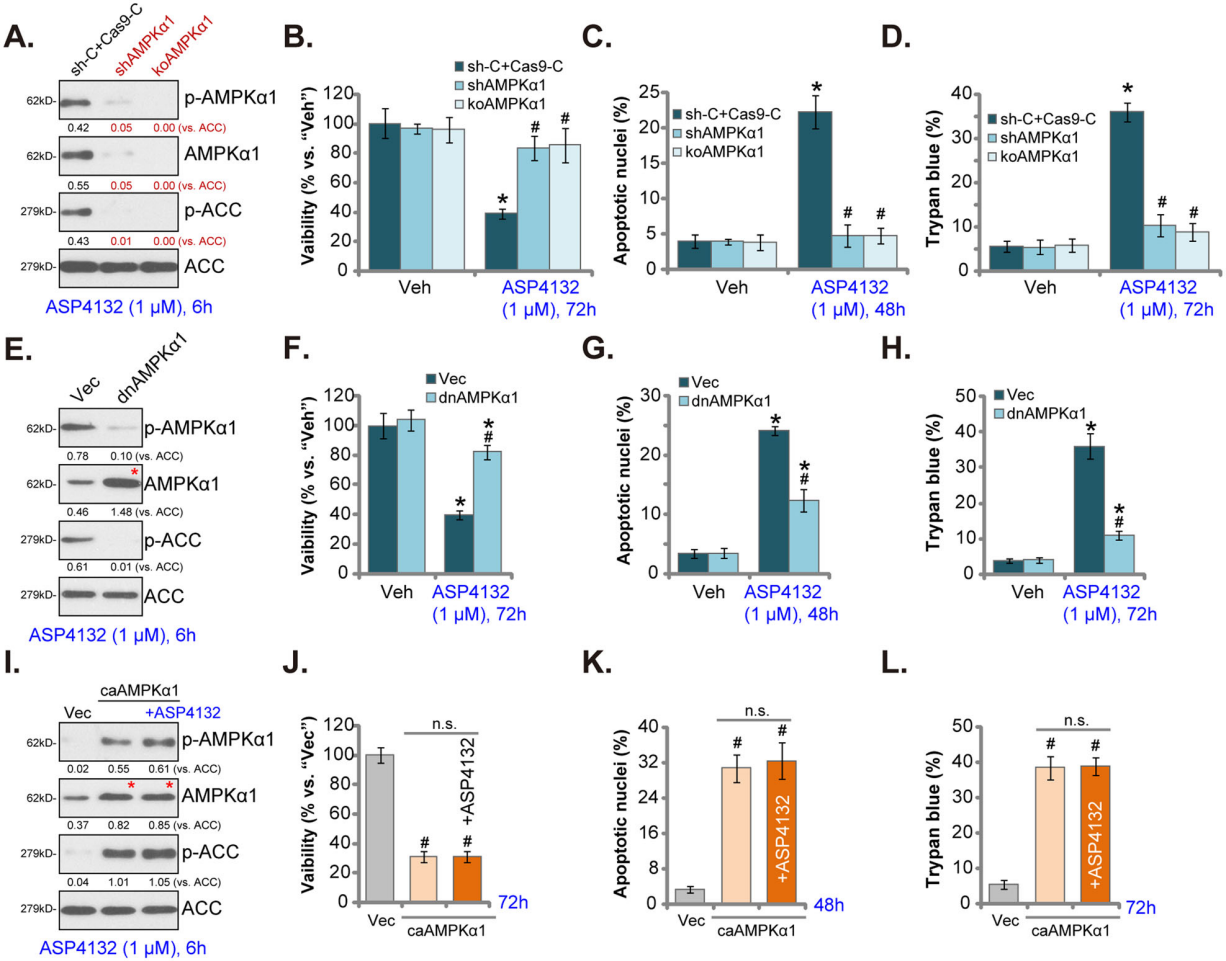
AMPK activation mediates ASP4132-induced anti-NSCLC cell activity. Stable pNSCLC-1 cells, expressing the lentiviral AMPKα1 shRNA (“shAMPKα1”), the CRISPR/Cas9-AMPKα1-KO construct (“koAMPKα1”) (**A**–**D**), the dominate negative AMPKα1 (T172A, “dnAMPKα1”) (**E**–**H**), the constitutively active AMPKα1 (T172D, caAMPKα1) construct (**I**–**L**), or corresponding control shRNA or empty construct, were established; Cells were treated with ASP4132 (1 μM) or vehicle control and cultured for applied time periods, expression of listed proteins was tested by Western blotting assays (**A**, **E**, **I**). Cell viability, apoptosis and death were tested by CCK-8 (**B**, **F**, **J**), apoptotic nuclei staining (**C**, **G**, **K**) and Trypan blue staining (**D**, **H**, **L**) assays, respectively. Expression of listed proteins was quantified and normalized to the loading control (**A**, **E**, **I**). Red stars indicated expression of the mutant AMPKα1 (**E**, **I**). “Veh” stands for the vehicle control (Saline). Data were presented as mean ± standard deviation (SD, n = 5). “sh-C + Cas9-C” stands for control pNSCLC-1 cells with scramble control shRNA plus CRISPR/Cas9 empty vector (**A**–**D**). “Vec” stands for empty vector (**E**–**L**). *p < 0.05 vs. “Veh” cells. ^**#**^*p* < 0.05 vs^.^ ASP4132-treatment in “sh-C + Cas9-C” control cells or “Vec” control cells. “n.s.” stands for non-statistical difference. The experiments were repeated five times with similar results detected.

To test AMPK activation is the primary reason of ASP4132-induced anti-NSCLC cell activity, lentiviral AMPKα1 shRNA was transduced to pNSCLC-1 cells. Stable cells were established following selection by puromycin: shAMPKα1 cells. In addition a CRISPR/Cas9-AMPKα1-KO construct was transfected to pNSCLC-1 cells. Single stable cells were established following GFP sorting plus puromycin selection: koAMPKα1 cells. As shown, AMPKα1 protein expression was depleted in both shAMPKα1 cells and koAMPKα1 cells (Fig. 5A). ASP4132-induced AMPK activation, or AMPKα1-ACC phosphorylation, was blocked in AMPKα1-silenced and AMPKα1-KO pNSCLC-1 cells (Fig. 5A). Significantly, ASP4132-induced viability (CCK-8 OD) reduction (Fig. 5B), cell apoptosis (increase of apoptotic nuclei ratio, Fig. 5C) and death (Fig. 5D) were reversed in shAMPKα1 cells and koAMPKα1 cells. Therefore, AMPK inhibition reversed ASP4132-induced anti-NSCLC cell activity, suggesting that AMPK activation was required for ASP4132-induced anti-NSCLC cell activity.

Next, a dominant negative AMPKα1 (T172A, dnAMPKα1^27,57^) was transduced to pNSCLC-1 cells, and stable cells were established: dnAMPKα1 cells. As shown the construct, dnAMPKα1, largely inhibited ASP4132-induced AMPKα1-ACC phosphorylation in pNSCLC-1 cells (Fig. 5E). Consequently, ASP4132-induced viability reduction (Fig. 5F), cell apoptosis (Fig. 5G) and death (Fig. 5H) were significantly attenuated in dnAMPKα1 cells (Fig. 5F–H). These results further supported that AMPK activation mediated ASP4132-induced anti-NSCLC cell activity.

To further support our hypothesis, a constitutively active AMPKα1 (T172D, caAMPKα1^35,58,59^) was stably transfected to pNSCLC-1 cells, resulting in robust increase of AMPKα1-ACC phosphorylation (Fig. 5I). Mimicking ASP4132-induced actions, caAMPKα1 induced significant anti-NSCLC cell activity, causing viability reduction (Fig. 5J), cell apoptosis (Fig. 5K) and death (Fig. 5L). Importantly, in caAMPKα1-expressing pNSCLC-1 cells adding ASP4132 failed to further increase AMPKα1-ACC phosphorylation (Fig. 5I). Neither did it produce further anti-pNSCLC-1 cell activity (Fig. 5J–L). Thus, ASP4132 was invalid on cell behaviors in caAMPKα1 NSCLC cells, further supporting that AMPK activation is the primary reason of ASP4132-induced anti-NSCLC cell activity.

### Oral administration of ASP4132 inhibits NSCLC xenograft growth in SCID mice

**Fig. 6:**
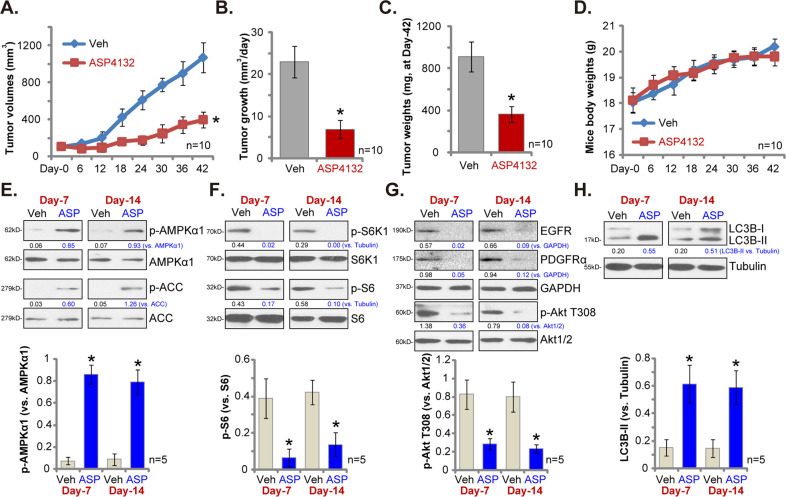
Oral administration of ASP4132 inhibits NSCLC xenograft growth in SCID mice. The SCID mice-bearing pNSCLC-1 xenograft tumors were treated with ASP4132 (oral administration, 5 mg/kg body weight, daily for 21 days) or vehicle control (ten mice per group/*n* = 10); Estimated tumor volumes (**A**) and mice body weights (**D**) were recorded every six days for a total of 42 days; Estimated daily tumor growth was calculated using the described formula (**B**); At the end of experiments, Day-42, tumors of the two groups were separated via surgery and weighted individually (**C**). At experimental Day-7 and Day-14, 4 h after ASP4132/vehicle administration, one tumor of each group was isolated and tumor tissues were subjected to Western blotting assay of listed proteins (**E**–**H**). Expression of listed proteins was quantified (**E**–**H**). Data were presented as mean ± standard deviation (SD).

Next we tested whether ASP4132 could inhibit NSCLC cell growth in vivo. As described, three weeks after pNSCLC-1 cells injection to SCID mice flanks, tumor xenografts were established (“Day-0”). Mice were then randomly assigned into two groups, receiving ASP4132 or vehicle administration. By recording tumor volumes we found that oral administration of a single dose of ASP4132 (5 mg/kg body weight, for 21 days) largely inhibited NSCLC xenograft growth in SCID mice (Fig. 6A). This concentration was based on the recommendation from the supplier. Volumes of NSCLC xenografts with ASP4132 administration were significantly lower than those of vehicle control xenografts (Fig. 6A). We next applied a formula to calculate the estimated daily tumor growth: (Tumor volume at Day-42—Tumor volume at Day-0)/42. Results showed that ASP4132 oral administration potently suppressed NSCLC xenograft growth in SCID mice (Fig. 6B). At Day-42 all tumors of the two groups were separated through surgery and weighted individually. Results in Fig. 6C demonstrated that ASP4132-treated NSCLC xenografts were dramatically lighter than the control tumors. Importantly, the mice body weights were not significantly different between the ASP4132 group and vehicle control group (Fig. 6D), indicating that mice should be well-tolerated to ASP4132 treatment regimen, and we did not detect any apparent toxicities.

At experimental Day-7 and Day-14, 4 h after initial ASP4132/vehicle administration, one tumor of each group was separated via surgery. Each tumor was cut into five small pieces and lysed separately in the tissue lysis buffer. Western blotting assays were carried out to test signaling proteins. As shown p-AMPKα1 and p-ACC levels were significantly elevated in ASP4132-stimualted NSCLC xenograft tissues (Fig. 6E), indicating AMPK signaling activation. To support mTORC1 inhibition in vivo, we found that p-S6K1 and p-S6 were largely inhibited in NSCLC xenograft tissues with ASP4132 administration (Fig. 6F). Furthermore, ASP4132 induced EGFR and PDGFRα protein degradation as well as Akt inhibition in NSCLC xenograft tissues (Fig. 6G). Moreover, LC3B-I to LC3B-II conversion in ASP4132-treated NSCLC xenograft tissues suggested autophagy activation in vivo (Fig. 6H). Quantitative analyses integrating all blotting data testing five samples per tumor (*n* = 5) confirmed that AMPKα1 phosphorylation, p-S6 inhibition, p-Akt inhibition and LC3B-II conversion were significant in tumor tissues with ASP4132 administration (Fig. 6E–H, lower panels). Therefore, in consistent with in vitro signaling findings, ASP4132 administration induced AMPK activation, mTORC1 inhibition, RTKs (EGFR and PDGFRα) degradation and downstream Akt inactivation, as well as autophagy induction in NSCLC xenograft tissues.

## Discussion

Lung cancer is still one of the leading causes of cancer-associated mortalities, as it accounts for almost one-fifth of all cancer death worldwide^1,2,60^. Of all lung cancer, 85% of them are NSCLC^60^. One primary reason of poor prognosis of NSCLC is the overwhelming resistance to current therapies^61–64^. Therefore, the development of novel and more efficacious therapeutics against this devastating disease is urgent^61–64^.

Several other AMPK activators were reported to exert potent anti-NSCLC cell activity, including cordycepin^18,65^, polyphyllin I^66^, baicalin^67^ and metformin^19,68^. These tested AMPK activators, howerever, required high concentrations (over 10–100 μM in vitro) to induce significant anticancer activities^18,65–67^. Poor water solubility and potential off-target cytotoxicity might limit its application in vivo^18,65–67^. Here in cultured primary NSCLC cells (pNSCLC-1/-2/-3) and established cell lines (A549 and NCI-H1944) a low concentration of ASP4132 (1 μM) can potently inhibited cell growth, viability and proliferation as well as cell migration and invasion (Fig. 1). In addition, robust cell cycle arrest and apoptosis activation were detected in ASP4132-treated NSCLC cells (Figs. 1–3). In vivo, oral administration of a single dose of ASP4132 largely inhibited NSCLC xenograft growth in SCID mice without inducing apparent toxicity (Fig. 6). Therefore, ASP4132 is a novel and potent anti-NSCLC cell agent.

Due to mutation, amplification, deletion, methylation and post-translational modifications, mTOR overactivation in NSCLC is essential for tumorigenesis, cancer progression and therapy resistance, and is often associated with poor prognosis^69,70^. Wang et al., found that SH2B1, a member of the SH2-domain containing family, activated mTOR signaling to promote NSCLC cell proliferation^71^. Kumar et al., found that PI3K-mTOR inhibition by antroquinonol inhibited NSCLC cell proliferation^72^. mTOR-driven cancer progression can be inhibited by activation of AMPK^73–75^. Activated AMPK is able to phosphorylate and activate TSC2 (Tuberous sclerosis protein 2) to block mTORC1 activation^50^. In addition AMPK phosphorylates and in-activates Raptor (regulatory associated protein of mTOR) to further inhibit mTORC1^76^. In the present study we showed that ASP4132 potently inhibited mTORC1 activation and blocked S6K1-S6 phosphorylation in NSCLC cells (Fig. 4). Furthermore, mTORC1 inactivation was detected in ASP4132-treated NSCLC xenograft tumor tissues (Fig. 6). Therefore, mTOR inactivation might be one important reason to explain ASP4132-induced anti-NSCLC cell activity.

Autophagy activation could play a positive and negative role in NSCLC progression^77^. The general idea is that the persistent activation of autophagy shall be able to induce autophagic cell death in NSCLC cells^77,78^. Activated AMPK directly associates with and phosphorylates the autophagy-initiating kinase Ulk1, which is the most important upstream component of the autophagy machinery^52,53,79^. Moreover, activated AMPK phosphorylates and activates TSC2 to suppress mTORC1 activation, leading to autophagy induction^50,51^. Jang et al., found that AMPK activation is required for autophagosome maturation and lysosomal fusion^80^. Here we found that AMPK activation by ASP4132 induced autophagy activation, causing LC3B-I to LC3B-II conversion and LC3B-GFP puncta formation in NSCLC cells (Fig. 4). LC3B-I to LC3B-II conversion was detected in NSCLC xenograft tumor tissues with ASP4132 administration as well (Fig. 6). These results implied that autophagy activation could actively participate in ASP4132-induced anti-NSCLC activity.

Besides apoptosis, programmed necrosis is another form of active cell death that occurs in cancer cells facing different stimuli. In colorectal cancer cells PF-543, a novel sphingosine kinase 1 (SphK1) inhibitor, provoked programmed necrosis by inducing MMP reduction and mitochondrial p53-CyPD complexation^49^. Qin et al., reported that salinomycin-induced anti-glioma cell activity was associated with programmed necrosis cascade activation^41^. Zhang et al., found that berberine-induced cytotoxicity in prostate cancer cells was mainly due to activation of CyPD-dependent programmed necrosis pathway^43^. In the present study, we found that ASP4132 induced programmed necrosis (together with apoptosis) in NSCLC cells, causing mitochondrial p53-CyPD-ANT1 association, mitochondrial depolarization and medium LDH release (Fig. 3). Significantly, CyPD shRNA or inhibition (CsA) alleviated ASP4132-induced cytotoxicity in NSCLC cells (Fig. 3). Therefore, concurrent activation of two independent cell death cascades, apoptosis and programmed necrosis, could explain the superior anti-NSCLC cell activity by this novel AMPK activator. The detailed mechanisms of programmed necrosis activation by the AMPK activator warrant further studies.

Chen et al., have demonstrated that forced activation of AMPK could induce degradation of multiple RTKs (PDGFRα, PDGFRβ and EGFR) by promoting their lysosomal translocation^27^. mRNA expression of these RTKs was however unchanged^27^. Here we displayed that ASP4132 treatment similarly induced PDGFRα-EGFR protein degradation and inhibited downstream Akt activation in NSCLC cells and xenograft tumor tissues (Figs. 4 and 6). Considering the critical role of RTK-Akt activation in NSCLC progression^69,70^, targeting this cascade by ASP4132 might be another reason of its superior anti-NSCLC cell activity.

In the current study the findings from in vitro assays and animal studies cannot be directly translated to humans. The safety and the anti-NSCLC efficiency of ASP4132 should be further tested. The respective contribution of apoptosis, programmed necrosis and autophagy to ASP4132-induced NSCLC cell death, as well as their relationships, warrant further investigations.

## Conclusion

Activation of AMPK by ASP4132 potently inhibits NSCLC cell growth in vitro and in vivo.

## Supplementary information

Supplementary Figure 1
