# Supplementary material for: AMPK activation by ASP4132 inhibits non-small cell lung cancer cell growth
Source: Cell Death Dis. 2021 Apr 6;12(4):365. doi: 10.1038/s41419-021-03655-2 (PMC8024326; doi:10.1038/s41419-021-03655-2)
Supplement: Supplementary file 1 — Supplementary Figure 1 [file 41419_2021_3655_MOESM1_ESM.pdf]

Figure S1

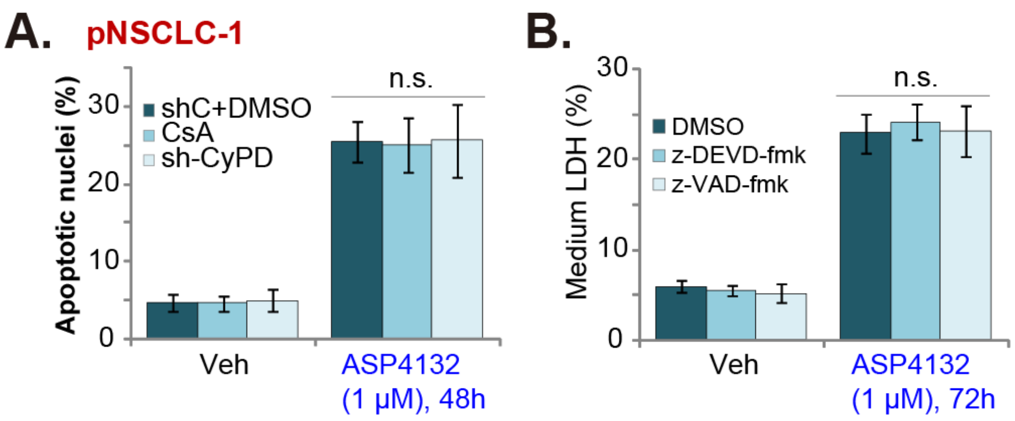

Figure S1. Stable pNSCLC-1 cells with CyPD shRNA lentiviral particles (sh-CyPD) or control cells with cyclosporin A pretreatment (CsA, 5 μ M, 1h pretreatment), as well as cells with vehicle control treatment plus scramble control shRNA lentiviral particles (“shC+DMSO”), were treated with ASP4132 (1 μM) and cultured for 48h, cell apoptosis was tested by apoptotic nuclei staining assay (results were quantified, **A**). pNSCLC-1 cells were pretreated with the caspase-3 inhibitor z-DEVD-fmk (50 μM, 1h pretreatment) or the pan caspase inhibitor z-VAD-fmk (50 μM, 1h pretreatment), followed by ASP4132 (1 μM) treatment and cultured for 72h, cell necrosis was quantified by medium LDH release assay (**B**). Data were presented as mean ± standard deviation (SD, n=5). “n.s.” stands for no statistical difference. The experiments were repeated five times with similar results detected.
